# PERCEPTIONS OF NURSES DELIVERING NURSING HOME VIRTUAL CARE SUPPORT: A QUALITATIVE PILOT STUDY

**DOI:** 10.1093/geroni/igac059.2574

**Published:** 2022-12-20

**Authors:** Justin Blackburn, Carol Mills, Yvette Tran, Valerie Yeager, Kathleen Unroe, Ann Holmes

**Affiliations:** IU Richard M. Fairbanks School of Public Health at IUPUI, Indianapolis, Indiana, United States; IU Richard M. Fairbanks School of Public Health at IUPUI, Indianapolis, Indiana, United States; IU Richard M. Fairbanks School of Public Health at IUPUI, Indianapolis, Indiana, United States; IU Richard M. Fairbanks School of Public Health at IUPUI, Indianapolis, Indiana, United States; Indiana University Center for Aging Research, Regenstrief Institute, Inc., Indianapolis, Indiana, United States; IU Richard M. Fairbanks School of Public Health at IUPUI, Indianapolis, Indiana, United States

## Abstract

Avoidable hospitalizations among nursing home residents result in poorer health outcomes and excess costs. Consequently, efforts to reduce avoidable hospitalizations have been a priority over the recent decade. However, many potential interventions are time-intensive, require dedicated clinical staff, and nursing homes are chronically understaffed. The Optimizing Patient Transfers, Impacting Medical Quality, and Improving Symptoms: Transforming Institutional Care (OPTIMISTIC) project was one of seven sites selected by CMS as "enhanced care & coordination providers" and was implemented from 2012 to 2020. A virtual program based on the principles of OPTIMISTIC was developed in the spring of 2020 with the goal of expanding the reach of the program’s services. This qualitative study explores the perceptions and experiences of the nurses that piloted a virtual care support project in 11 nursing homes in a midwestern state, and identified the nurses’ perceived facilitators of, and barriers to, the effectiveness of delivering a novel virtual care support program. A key finding from this analysis is that relationships, communication, and access to information were identified as common themes facilitating or impeding the perceived effectiveness of implementation of virtual care support programs within nursing homes, from the perspective of the nurses delivering the services. The experiences and recommendations of the program nurses provide insights into crucial elements important to the implementation of similar virtual care support models, and the role of telehealth in bridging healthcare workforce gaps.

